# Effects of a tailored strength training program of the upper limb combined with transcranial direct current stimulation (tDCS) in chronic stroke patients: study protocol for a randomised, double-blind, controlled trial

**DOI:** 10.1186/s13102-019-0120-1

**Published:** 2019-05-24

**Authors:** Marie-Hélène Milot, Stephania Palimeris, Hélène Corriveau, François Tremblay, Marie-Hélène Boudrias

**Affiliations:** 1 Faculté de médecine et des sciences de la santé, École de réadaptation, Université de Sherbrooke, 1036 Belvédère sud, Sherbrooke, Québec J1H 4C4 Canada; 2 Faculty of Medicine, School of Physical and Occupational Therapy, McGill University, Montreal, Quebec Canada; 3 Feil and Oberfeld Research Centre, BRAIN Lab, Jewish Rehabilitation Hospital, Laval, Quebec Canada; 4 Montreal Center for Interdisciplinary Research in Rehabilitation (CRIR), Montreal, Quebec Canada; 5 Faculty of Health Sciences, School of Rehabilitation Sciences, University of Ottawa, Ottawa, Ontario Canada

**Keywords:** Stroke, Exercise, Transcranial magnetic stimulation, Randomised controlled trial, Transcranial direct current stimulation, Upper limb

## Abstract

**Background:**

A significant proportion of individuals are left with poor residual functioning of the affected arm after a stroke. This has a great impact on the quality of life and the ability for stroke survivors to live independently. While strengthening exercises have been recommended to improve arm function, their benefits are generally far from optimal due to the lack of appropriate dosing in terms of intensity. One way to address this problem is to develop better tools that could predict an individual’s potential for recovery and then adjust the intensity of exercise accordingly. In this study, we aim at determining whether an individualized strengthening program based on the integrity of the corticospinal tract, as reflected in the amplitude of motor evoked potentials (MEPs) elicited by transcranial magnetic stimulation (TMS), in conjunction with transcranial direct current stimulation (tDCS), could lead to more optimal outcomes in terms of arm function in chronic stroke patients.

**Methods:**

This multicentre, double-blinded, randomised controlled trial will aim to recruit 84 chronic stroke patients. Before and after training, participants will undergo a clinical evaluation, assessing motor recovery of the affected arm (Fugl-Meyer Stroke Assessment-FMA) and a TMS evaluation to assess the integrity of the corticospinal tract, as reflected in MEP amplitude. Based on their baseline MEPs amplitude, participants will be stratified into three groups of training intensity levels determined by the one-repetition maximum (1RM); 1) low: 35–50% 1 RM (MEPs < 50 μV); 2) moderate: 50–65% 1RM (MEPs 50-120 μV); and 3) high: 70–80% 1RM (MEPs > 120 μV). Training will target the affected arm (3 times/week for 4 weeks). In addition, participants will be randomly allocated into two tDCS groups (real vs. sham) and tDCS will be applied in an anodal montage during the exercise.

**Discussion:**

This study will determine whether an individualized strength training intervention in chronic stroke survivors can lead to improved arm function. In addition, we will also determine whether combining anodal tDCS over the lesioned hemisphere with strength training can lead to further improvement in arm function, when compared to sham tDCS.

**Trial registration:**

ClinicalTrials.gov Identifier: NCT02915185. Registered September 21 2016.

## Background

Stroke is a leading cause of severe long-term disability across the globe [1, 2]. One of the most common disabling consequences of stroke is residual muscle weakness or paresis of the affected arm [3, 4], which has a significant impact on patients’ activities of daily life and is a major contributor to reduced quality of life [5, 6]. Consequently, there has been a move to implement strength training as part of rehabilitation after stroke [7], to increase strength and improve function of the affected arm, even at the chronic stage where gains are still observed [8]. Strength training is commonly used with progressive resistance or repetitive practice and there is evidence demonstrating that it is an effective intervention to improve strength and activity after stroke [7, 9]. Yet, variability is observed between individuals, in which some patients demonstrate significant gains in response to strength training programs while others show either minimal or no benefits [3, 10]. This brings to question whether the intensity of strength training programs is sufficient to produce a training stimulus and challenge individuals’ maximum capacity. Although there is a variety of strengthening protocols prescribed to stroke patients, few studies in the rehabilitation literature assess critical training parameters to address each individual’s needs and impairments [11, 12]. A meta-analysis by Coupar et al. [13] found that neurophysiological factors, such as the integrity of the corticospinal tract assessed by non-invasive brain stimulation (NIBS) techniques, were strongly associated with upper limb recovery after a stroke; supporting the use of neurophysiological markers in determining a person’s potential for recovery and functional performance. Thus, there is an urgent need to identify valid biomarkers of recovery to design better training interventions for the management of post-stroke disability, notably by adapting programs to meet each individual’s capacity in terms of potential for recovery.

Transcranial magnetic stimulation (TMS) is a NIBS technique allowing the identification of biomarkers reflecting the integrity of the corticospinal system after a hemispheric stroke [14]. Stimulation of the motor cortex can elicit motor evoked potentials (MEPs) in contralateral limb muscles, whose presence after a stroke is strongly suggestive of preserved functional projections and potential for recovery in the affected limbs. For instance, Jo et al. [15] reported that the presence of MEPs from both hand motor cortices in the early subacute phase was a good predictor of motor function in patients at 3 months after stroke onset. The combining use of TMS and tractography on 53 patients with intracerebral hemorrhage and severe motor weakness showed that patients in whom MEPs could be elicited in the paretic upper limb, and with a preserved corticospinal tract, had better motor outcomes at 6 months post-stroke [16]. On the other hand, other studies have reported that absence of MEPs in response to high intensity TMS of the ipsilesional motor cortex was associated with poor motor recovery of the upper limb in both the acute and chronic stages post stroke [17–19]. In addition to their ability to predict motor recovery, MEPs can also provide information on an individual response to exercises [20]. Several studies have highlighted the importance of this measure not only to assess corticospinal tract integrity to predict patients’ potential for recovery but also to predict their response to exercise [18, 21, 22]. Stinear et al. [18] for example, proposed an algorithm that uses MEPs as a marker for stroke survivors’ stratification in terms of exercises prescription to optimise functional recovery. Among the various parameters included in the algorithm, such as DTI and the presence of movements at the thumb and shoulder joints, the absence/presence of MEPs in response to TMS from the ipsilesional motor cortex was considered as a crucial parameter to consider in order to stratify patients based on their recovery potential.

In parallel, recent developments in the management of stroke disability indicate that further gains in function can be obtained when rehabilitation interventions are combined with neurostimulation techniques designed to boost motor excitability and enhance response to exercises [23]. The modulation of cortical excitability by transcranial direct current stimulation (tDCS) has gained particular interest because of its promising effects in neurorehabilitation after stroke [24], which include change in cortical excitability [25–27], enhancement of motor performance and change of movement accuracy and speed [28, 29]. The technique aims at modulating neuronal excitability using a constant and weak current (1–2 mA) which passes through electrodes placed on the scalp. Depending on the current direction, the stimulation can either enhance (anodal) or depress (cathodal) the excitability of the stimulated cortical area [30]. A number of studies have investigated the impact of anodal tDCS on motor recovery of the affected upper limb of stroke patients [31]. Kim et al. [32] stimulated the ipsilesional cortical region of 10 subacute stroke patients on average 12 weeks post infarct and reported significant improvement in motor performance of the hemiparetic hand, outlasting the stimulation session for at least 60 min. Similar results were obtained by Fregni et al. [33] and Hummel et al. [34], where stroke patients were found to have functional improvement in the paretic hand that outlasted the stimulation period with an improvement magnitude of 6.7 and 8.9%, respectively. Furthermore, results from a meta-analysis [35] also support the therapeutic potential of tDCS as an adjuvant treatment strategy to enhance training in stroke patients with upper limb deficits, where tDCS demonstrated a significant impact on rehabilitative training with a moderate effect size of + 0.52 (*p* < 0.001) and + 0.69 (*p* < 0.001) for both immediate and longer-lasting analyses (up to 6 months), respectively.

According to the existing literature, there is a clear need to further validate the use of MEPs as a classification tool and to explore new ways on how to refine the use of this measure, in order to optimize post-stroke training interventions. In addition, since tDCS is still relatively new in the field of stroke rehabilitation, no study to date has tried to determine whether tDCS can enhance the effects of a strength training intervention in stroke patients; a type of intervention commonly used in rehabilitation [3]. Ultimately, there is an urgent need to design better training interventions for the management of post-stroke disability, notably by adapting programs to meet each individual’s capacity in terms of potential for recovery. There is also an important need to determine whether non-invasive brain stimulation techniques, such as tDCS, can be used in conjunction with tailored strength training exercises to boost functional recovery after stroke.

The primary goal of this RCT is to determine whether a 4-week tailored strength training program could lead to improved arm function in chronic stroke survivors. The secondary aim is to determine whether combining anodal tDCS of the lesioned hemisphere with strength training could lead to further improvement in arm function when compared to sham tDCS. This study will help determine whether tailored strength training interventions in stroke survivors, based on MEPs amplitude, can lead to greater gains in arm function. In addition, it will also establish whether combining anodal tDCS with tailored strength training can further promote recovery in stroke survivors when compared to sham tDCS.

## Methods

### Study design and setting

**Fig. 1:**
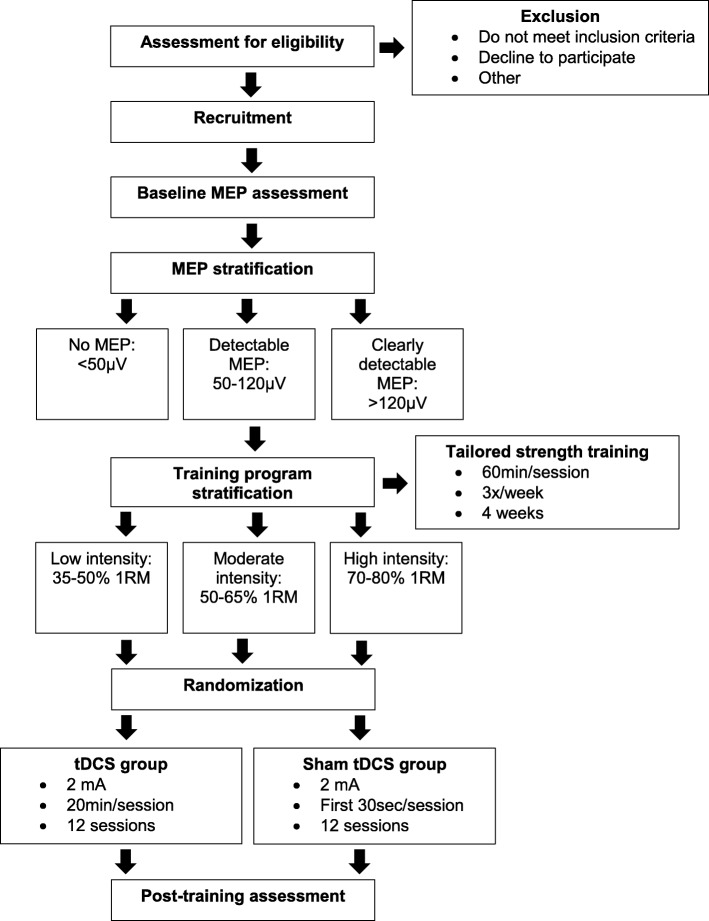
Study design flow diagram

This is a multi-centre, randomised controlled trial (RCT) study. Figure 1 illustrates the flow diagram of the study from recruitment and screening for eligible participants to post-training outcome assessments. The RCT takes place in Canada and involves three recruiting sites in two provinces (Québec: Montréal, Sherbrooke; Ontario: Ottawa). The study is ongoing and is currently during subject recruitment phase (May 2017 till present).

### Participants

Participants will be recruited using several recruitment procedures such as newspaper advertisement, search in medical archives and former patient lists from each site. Potential participants will first be screened over the phone to determine their interest and initial eligibility to participate in the study following a script for early recruitment, after which they will be invited to attend each site, for a screening visit and evaluation. Screening and clinical evaluations will be performed to verify eligibility to participate. At the beginning of the first study visit, written consent will be obtained from each participant and all participants will be informed about the duration and procedure of the study.

#### Inclusion/exclusion criteria

Males and females will be eligible to participate in the study if they meet the following criteria: 1) are aged 18 years and older; 2) have had solely one stroke; 3) be in a chronic stroke phase (> 6 months) and 4) have completed their rehabilitation treatment. Participants will be excluded from the study if any of the following criteria are met: 1) a significant spasticity at the affected upper limb (a score ≥ 3 on the *modified Ashworth scale*) [36]; 2) a significant pain intensity at the affected upper limb (a score ≥ 6/10 on the Visual Analog Pain Scale) [37]; 3) a major sensory deficit (a score ≤ 25/34 on the *Nottingham Sensory Assessment*) [38]; 3) a presence of hemineglect (> 70% of unshaded lines on the same side as the motor deficit on the *Line Cancellation Test*) [39]; 4) an apraxia (a score > 2.5 on the *Alexander Test*) [40]; 5) any cognitive impairments (a score ≤ 2/5 on the *Mini-Cog Test*) [41]; 6) the presence of a neurological disorder other than a stroke; 7) concomitant orthopaedic problems at the affected upper limb and 8) any contraindication to TMS and/or tDCS, such as epilepsy, metallic implants, a cardiac pace-maker or pregnancy, for female subjects.

### Assessment period

Prior to and following the intervention period, participants will complete clinical and neurophysiological evaluations. Each evaluation day will last approximately 1.5 h and both visits will take place in the week before (baseline) and the week after (post-training) the intervention.

#### Randomisation and blinding

According to the amplitude of their TMS-induced MEP responses, three levels of stratification will be used, which are adapted from Milot et al. [22]: 1) no detectable MEPs (< 50 μV); 2) detectable MEPs (50-120 μV) and 3) clearly detectable MEPs (> 120 μV). Within each stratum, participants will be further randomly allocated into two tDCS groups: 1) tDCS real group and 2) tDCS sham group. Participants will be randomly assigned to either the tDCS real group or the tDCS sham group with a 1:1 allocation. Randomisation allocation will be generated by a computer using a blocked randomisation with variable block sizes of 2 and 4. The participants, the evaluators, involved in the clinical and neurophysiological evaluations, the research staff enrolling participants and the data analysts will be blinded to the tDCS group assignment.

#### Clinical evaluation

Baseline participant information including age, gender, time since stroke and location of stroke will be collected at the initial visit of each participant by a blinded evaluator to help characterize the sample. The clinical evaluations will consist of measurement of the range of motion of shoulder flexion, elbow flexion and wrist extension of both upper limbs, the Fugl-Meyer Stroke Assessment-FMA (upper limb section; 66 = normal) [42], the Box and Block Test [43], the Motor Activity Log [44] and measurement of grip strength, measured by the JAMAR® hand dynamometer.

#### Neurophysiological evaluation

The neurophysiological evaluation will be performed by a trained evaluator blinded to the participants’ tDCS group allocation and will consist of assessment of the integrity of the corticospinal tract and cortical reorganization post training through the use of motor evoked potentials (MEPs) elicited by TMS. For TMS testing, participants will be seated in an armchair with both hands resting in pronation. The participant’s skin will be cleaned with alcohol and surface electromyography (EMG) electrodes will be positioned over the first dorsal interosseous (FDI) of both hands and the extensor carpi radialis (ECR) of the affected arm. Using a TMS system (Magstim 200^2^, Magstim Company, Dyfed, UK) and a 70-mm figure-of-eight coil, we will first determine the hotspot and resting motor threshold (rMT, lowest intensity to evoke reliable MEPs ≥50 μV) from the contralesional hemisphere. These coordinates will be used to guide the identification of the optimal location of the ipsilesional primary motor cortex (M1) of the affected hemisphere, when needed. The rMT will then be determined using the Motor Threshold Assessment Tool software (MTAT 2.0; Clinical Researcher, Knoxville, TN, USA). The software allows for fast estimation of motor threshold through the maximum-likelihood strategy based on the PEST (Parameter Estimation by Sequential Testing) algorithm [45]. Next, a series of suprathreshold stimuli (130% rMT, *n* = 10) will be delivered over the hand motor area to elicit MEP in the resting state. We will repeat these procedures for the ipsilesional hemisphere. If no MEPs can be elicited in the affected FDI, the coil will be moved to target the representation of the arm to elicit MEPs in the affected ECR. If peak-to-peak MEP amplitude of the affected ECR does not reach the chosen threshold of 50 μV, even at the maximum output of the stimulator (100%), the participant’s response will be classified as “MEP absent”. After testing in the resting state, corticomotor excitability of the lesioned hemisphere will be tested in the active state. Participants will remain seated in the armchair and will be asked to actively exert a constant force (20% of maximal voluntary effort depending on the participant’s ability to contract) against a pinch dynamometer on the affected side using a lateral key-pinch with the thumb and index finger. During the contraction (duration 5 s), a suprathreshold TMS pulse (130% of rMT) will be delivered at 3 s to elicit a facilitated MEP along with a silent period (SP). The SP refers to the interruption of EMG activity in the target muscle arising from spinal and cortical inhibition (GABA B receptors) resulting from the stimulation [46]. Both facilitated MEPs and SP will be measured 5 times with at least 30 s between trials to allow for recovery.

### Intervention period

All subjects will participate in an outpatient supervised tailored strength training program, which will follow the recommendations of the American Stroke Association (ASA) position on exercise prescription after stroke [47]. Specifically, it will be a 4-week training intervention, performed 3 times per week non-consecutively, with 3 series per exercise of 10 repetitions and a 2-min break in between exercises. The strength training program will be conducted for 1 h in an outpatient rehabilitation setting and supervised by an experienced trainer who will closely monitor the participants’ exercise performance. The training will commence with a 5-min warm-up comprising of active movements of the muscles to be trained. Using free weights, the one-repetition maximum (1RM), which is the maximum amount of weight load an individual can lift for one repetition, will be estimated by the 10RM [48] in order to avoid tendino-muscular injuries and fatigue. The 10RM will be determined for the muscles playing a key role in the functional performance of the upper limb [49, 50]. These muscles are the wrist extensors and the elbow and shoulder flexors. In addition, the grip muscles of the affected hand will be trained with a JAMAR® dynamometer. Participants’ maximal grip force will be determined and used to dose the training of the hand muscles and its progression.

As intensity plays a crucial role in response to training, the intensity of the strength training program will be tailored to each participant’s potential for recovery based on their baseline MEP amplitudes and gradation of the intensity will follow the ASA recommendation [47] and will be based on the 1RM. This same gradation will be applied for the hand muscles based on the participants’ maximal grip force on the JAMAR®. Thus, for participants in the MEP < 50 μV strata (no detectable MEP; low potential for recovery), the training will start at 35% of the 1RM for each muscle group and will then be increased by 10% each week to reach 50% of the 1RM by week 4. For participants in the MEP 50-120 μV strata (detectable MEP; moderate potential for recovery), the training will start at 50% of the 1RM to reach 65% by week 4. For the participants in the MEP ≥120 μV strata (clearly detectable MEP; high potential for recovery), they will train at 70% of the 1RM during week 1 and progress to 80% of the 1RM at week 4. In combination to the % 1RM, the Borg Rating of Perceived Exertion Scale (BRPE), a measure of an individual’s self-perceived physical exertion during exercise, will be used to further control the intensity of exercise within and between MEP strata while taking into account the individual’s residual ability. The Borg Scale can be accurately rated by chronic stroke survivors and is a valid tool for regulating exercise intensity [51, 52]. Thus, for the first three training weeks, participants in each MEP stratum will train at a perceived intensity between 11 and 13/20 (“fairly light”/“somewhat hard”), corresponding to about 66% of an individual’s maximal effort, and progress toward a perceived intensity between 15 and 17/20 (“hard”/“very hard”), corresponding to about 80% of an individual’s maximal effort, at week 4.

#### Transcranial direct current stimulation (tDCS)

An anodal montage over the ipsilesional hemisphere will be used. The localisation of the lesioned M1 and placement of electrodes will follow the protocol from DaSilva et al. [53]. In essence, the vertex will be located by marking the distance halfway between the nasion and inion and the distance between the right/left pre-auricular points. Location of M1, corresponding to C3/C4 on the EEG system, will be estimated by using 20% of the pre-auricular distance from the vertex. The anode will be placed over the M1 area whereas the cathode will be placed on the contralateral supra-orbital region. For the tDCS group, a direct current will be generated by a tDCS stimulator and gradually increased in a ramp-like fashion over the first 8 s until a maximum intensity of 2 mA is achieved. The tDCS will be applied for 20 min during each training session for a total of 12 sessions. The parameters chosen in the proposed project are considered safe for the application of tDCS [30, 31, 54]. For the group receiving sham tDCS, the protocol will be similar to the tDCS real group although the stimulation will be applied for the first 30 s only; a duration long enough to induce similar perceived sensation as real tDCS, to ensure blindness of the participants to the tDCS type [55]. After each training session, using a home-developed questionnaire, participants will be questioned about the presence of tDCS symptoms, their intensity and relatedness to tDCS.

If any participants develop contra-indications to the training intervention or the tDCS stimulation, they will be discontinued from the intervention but will be included in an intention to treat analysis.

### Outcome measures

#### Primary outcome measures

The primary outcome measures will be change in Fugl-Meyer Stroke Assessment Scale-FMA (66 = normal) [42], which will be used to assess changes in the trained arm motor function and change in peak-to-peak MEP amplitude, motor threshold and silent period, elicited by TMS, to assess changes in motor cortex excitability and cortical reorganization.

#### Secondary outcome measures

Secondary outcome measures will include:

- change in grip strength, which will be measured by the JAMAR® hand dynamometer (average of 3 trials in kg);
- change in Box and Block test, which will be used to evaluate manual dexterity by counting the number of blocks that can be moved from one compartment to another in 60 s [43];
- change in Motor Activity Log, which will assess participants’ self-reported level and quality of use of the affected arm in activities of daily living (ADL) [44] and;
- change in active and passive ranges of motion in shoulder flexion, elbow flexion and wrist extension, which will be assessed with a goniometer.

Along with the FMA, these variables were chosen to ensure that the most severely affected participants would be able to perform to some extent the required tasks, knowing that individuals without MEPs often present limited voluntary movement at the affected upper limb.

### Sample size and power calculations

A priori power analysis was performed in G*Power 3.1.9.2, using a two-tailed independent samples t-test having an alpha level of 0.05, to calculate the sample size required in this study. By stratifying participants based on their MEP amplitude to provide an appropriate dosage of training, we expect all participants within the three MEP strata to benefit from the 4-week tailored strength training program. The sample size was thus calculated based on the expected difference in motor function gains between the tDCS real and the tDCS sham groups. Based on the results of studies having used repetitive application of tDCS in chronic stroke survivors, we expect an 8-point gain in FMA for the tDCS real group [56]. Furthermore, preliminary results based on this study showed a 7-point gain on the FMA scale after 4 weeks of strength training for participants presenting pre-training MEPs. Therefore, we estimate that the tDCS sham group (including participants with and without MEP) will show at least a 6-point gain on the FMA scale, exceeding the 5-point gain minimal detectable change (MDC) of this scale [57]. Thus, with an estimated average difference in FMA gain between both groups of 2 points (SD = 3), with an effect size of 0.66 and a power of 85%, we calculated that a total of 84 participants will be needed to detect differences between groups. However, presuming an attrition rate of 20%, a total of 105 participants will be recruited for the purpose of this study.

### Statistical analysis

Descriptive statistics will be used to characterize the sample. We will verify if, at the beginning, the two tDCS groups will be comparable by using independent t-tests or Chi-squared tests, depending on the nature of the variables. To evaluate the impact of tailored exercises based on MEP stratification on changes in FMA and MEP measures, as well as the impact of tDCS on enhancement of training response, a two-way 3X2 ANOVA [MEP strata (three levels) and type of tDCS (2 levels)] will be used. The significance level will be set at 0.05. If an interaction is noted, paired *t*-tests will be used to locate any significant differences in each stratum with a Bonferroni correction for multiple tests (adjusted *p*-value of 0.02).

## Discussion

This study will be the first RCT to integrate a commonly used rehabilitation treatment, which is a strength training program of the affected arm, with state-of-the-art brain evaluation protocols and neurostimulation techniques (TMS and tDCS) to allow optimization of the intensity of training based on each stroke survivor’s recovery potential. This study will create an initial, yet strong evidence base for MEP stratification as a tool to guide clinicians in providing optimal tailored exercise programs to support recovery post-stroke. More importantly, the marked expected improvement in the affected upper limb’s motor function, following the tailored strength training program, will allow stroke survivors to have a more active lifestyle and ultimately optimal quality of life.

## Abbreviations

ADL: Activities of daily living
ASA: American stroke association
BRPE: Borg Rating of Perceived Exertion Scale
ECR: Extensor carpi radialis
EMG: Electromyography
FDI: First dorsal interosseous
FMA: Fugl-Meyer Stroke Assessment Scale
M1: Primary motor cortex
MDC: Minimal detectable change
MEP: Motor evoked potential
NIBS: Non-invasive brain stimulation
RCT: Randomised controlled trial
RM: Repetition maximum
rMT: Resting motor threshold
SD: Standard deviation
SP: Silent period
tDCS: Transcranial direct current stimulation
TMS: Transcranial magnetic stimulation
